# Angiotensin II increases activity of the ClC-K2 Cl^−^ channel in collecting duct intercalated cells by stimulating production of reactive oxygen species

**DOI:** 10.1016/j.jbc.2021.100347

**Published:** 2021-01-30

**Authors:** Naghmeh Hassanzadeh Khayyat, Oleg Zaika, Viktor N. Tomilin, Kyrylo Pyrshev, Oleh Pochynyuk

**Affiliations:** Department of Integrative Biology and Pharmacology, The University of Texas Health Science Center at Houston, Houston, Texas, USA

**Keywords:** chloride transport, G protein-coupled receptor (GPCR), hypertension, ion channel, NADPH oxidase, renal physiology, Ang II, Angiotensin II, AQP2, aquaporin type 2, AT_1_R, Angiotensin receptor type 1, ENaC, epithelial Na^+^ channel, NOX, NADPH oxidase, *P*_*o*_, open probability, PI3-K, phosphoinositide 3 kinase, ROS, reactive oxygen species

## Abstract

The renal collecting duct plays a critical role in setting urinary volume and composition, with principal cells transporting Na^+^ and K^+^ and intercalated cells mediating Cl^−^ reabsorption. Published evidence implies Angiotensin II (Ang II) is a potent regulator of the collecting duct apical transport systems in response to systemic volume depletion. However, virtually nothing is known about Ang II actions on the basolateral conductance of principal and intercalated cells. Here, we combined macroscopic and single channel patch clamp recordings from freshly isolated mouse collecting ducts with biochemical and fluorescence methods to demonstrate an acute stimulation of the basolateral Cl^−^ conductance and specifically the ClC-K2 Cl^−^ channel by nanomolar Ang II concentrations in intercalated cells. In contrast, Ang II did not exhibit measurable effects on the basolateral conductance and on K_ir_4.1/5.1 potassium channel activity in principal cells. Although both Ang II receptors AT_1_ and AT_2_ are expressed in collecting duct cells, we show that AT_1_ receptors were essential for stimulatory actions of Ang II on ClC-K2. Moreover, AT_1_R^−/−^ mice had decreased renal ClC-K2 expression. We further demonstrated that activation of NADPH oxidases is the major signaling pathway downstream of Ang II-AT_1_R that leads to stimulation of ClC-K2. Treatment of freshly isolated collecting ducts with Ang II led to production of reactive oxygen species on the same timescale as single channel ClC-K2 activation. Overall, we propose that Ang II-dependent regulation of ClC-K2 in intercalated cells is instrumental for stimulation of Cl^−^ reabsorption by the collecting duct, particularly during hypovolemic states.

Hypertension is one of the major causes of morbidity and mortality affecting approximately 46% of US adults, with blood pressure in 50% of hypertensive individuals exhibiting a salt-sensitive pattern (1, 2). Elevated blood pressure is commonly caused by expansion of the circulating volume due to salt retention by the kidney (3). Variations in dietary salt intake regulate transport in the renal collecting duct *via* the renin–angiotensin–aldosterone system to shape urinary NaCl excretion and to maintain circulating volume (4, 5). The collecting duct is composed of electrically uncoupled principal and intercalated cells (6, 7). Principal cells perform electrogenic Na^+^ reabsorption *via* the epithelial Na^+^ channel (ENaC) localized to the apical membrane and the Na^+^/K^+^ ATPase on the basolateral membrane (5, 8, 9). Intercalated cells are essential for maintaining acid–base balance by secreting H^+^ *via* the apical V-ATPase (A-type) and HCO_3_^−^ *via* pendrin (SLC26A4) in the B-type (7). In addition, both A- and B-types have the capacity to reabsorb Cl^−^ even when ENaC activity is blocked with amiloride (10). Since both types of intercalated cells are involved, it is viewed that Cl^−^ reabsorption could occur with little or no changes in net acid or base secretion (11).

The long-standing paradigm suggests that Ang II-driven secretion of the mineralocorticoid aldosterone from adrenal gland leads to upregulation of the ENaC-dependent Na^+^ reabsorption in the collecting duct during the volume-depleted states (6). However, cumulative evidence demonstrates aldosterone-independent direct actions of Ang II on Na^+^ and Cl^−^ transport in the collecting duct during variations in salt intake and in the pathophysiology of Ang-dependent hypertension (10, 12, 13, 14). In fact, kidneys have substantial capacity to locally produce Ang II. In the experimental animal models of Ang II-induced hypertension (15, 16), intrarenal Ang II levels become much higher (over 100-fold) than those in plasma (17, 18, 19). Ang II binds to AT_1_ and AT_2_ receptors to exert its numerous physiological actions. Activation of AT_1_R promotes proliferation, vasoconstriction, antinatriuresis, salt appetite, etc. (20, 21, 22, 23, 24). AT_2_R antagonizes the actions of AT_1_R resulting in vasodilation, natriuresis, and prostaglandin release (20, 22, 25). Both AT_1_R (most abundantly AT_1a_R isoform in mice) and AT_2_R are expressed at the apical and basolateral sides of the collecting duct cells, although AT_2_R expression is considerably lower (25, 26, 27). Chronic Ang II infusion stimulates ENaC activity well above the physiological range of regulation (13), which cannot be effectively inhibited by mineralocorticoid receptor blockade (28). Ang II also increases Cl^−^ reabsorption, in part by stimulating apically localized HCO_3_^−^/Cl^−^ exchanger pendrin in B-type intercalated cells (10). At the same time, the actions of Ang II on the basolateral conductance of the collecting duct cells are not known.

Basolateral electrical conductance of the collecting duct principal cells is almost exclusively K^+^ selective (29, 30). The most prevalent heteromeric inward rectifying K_ir_4.1/5.1 40 pS potassium channel is essential for K^+^ recycling to set up a strong hyperpolarizing resting potential on the basolateral membrane around −70 mV to establish a favorable driving force for ENaC-mediated Na^+^ reabsorption (29, 31). K_ir_4.1/5.1 is also expressed in the upstream segments, most notably the distal convoluted tubule, to control NaCl reabsorption *via* thiazide-sensitive NCC cotransporter (32, 33). Loss-of-function mutations in the *KCNJ10* gene encoding K_ir_4.1 subunit result in EAST/SeSAME syndrome, a complex electrolyte imbalance disorder manifested as hypotension, natriuresis, hypocalciuria, hypomagnesemia, and hypokalemic metabolic alkalosis (34, 35). Consistently, *Kcnj16* deletion encoding the K_ir_5.1 subunit ameliorated the development of salt-sensitive hypertension in Dahl SS rats (36).

Intercalated cells of the collecting duct do not express Na^+^/K^+^ ATPase and have no electrogenic basolateral potassium conductance (7, 30). Instead, activity of the ClC-K2 chloride channel determines basolateral Cl^−^ transport and sets the resting potential around −20 mV (37, 38). Similarly to K_ir_4.1/5.1, ClC-K2 is also expressed in the distal nephron segments, namely, the thick ascending limb and distal convoluted tubule (37, 39). Inactivating mutations in the *CLCNKB* gene (encoding ClC-Kb, human version of ClC-K2) underlie Bartter’s syndrome type III associated with hypotension, hypochloremia, and metabolic alkalosis (40, 41, 42). Of note, K_ir_4.1/5.1 and ClC-K2 are expressed in all cells of the thick ascending limb and distal convoluted tubule, whereas they are separated to principal and intercalated cells of the collecting duct, respectively (43). It is possible that such mosaic architecture of the collecting duct allows independent cell type–specific regulation of Na^+^ and Cl^−^ transport by endocrine signals, such as Ang II.

The major focus of the current study was to explore the functional consequences and uncover the molecular mechanisms of Ang II actions on K_ir_4.1./5.1 potassium and ClC-K2 chloride conductance in native collecting duct cells.

## Results

### Ang II increases ClC-K2–dependent basolateral conductance in intercalated collecting duct cells

**Figure 1:**
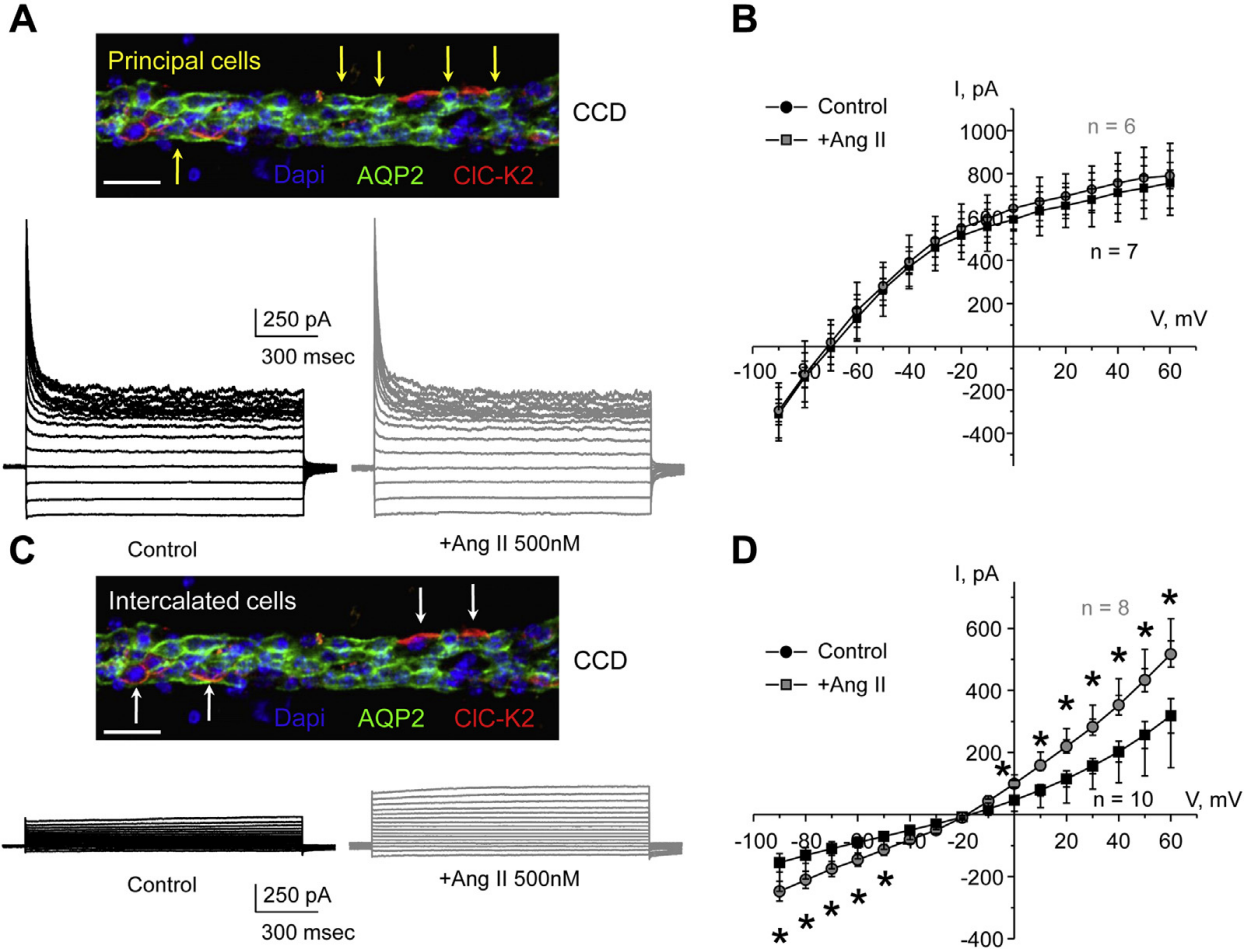
**Ang II increases basolateral chloride currents in intercalated cells of the collecting duct.***A*, representative macroscopic currents in individual principal cells in response to voltage steps from −90 to +60 from the holding potential of −60 mV in the control (*black*) and following treatment with Ang II (500 nM) for 3 min (*gray*). A micrograph of a typical isolated collecting duct shown on *top*. The expression of AQP2, a marker of principal cells (highlighted with *yellow arrows*), is shown with pseudocolor *green*. Nuclear marker DAPI is shown with pseudocolor *blue*. The scale bar represents 70 μM. *B*, current–voltage (I–V) relations of the basolateral K^+^-selective conductance obtained from voltage step protocols as shown in *A* in the control (*black*) and upon treatment with Ang II (*gray*). The number of individual recordings is shown. Measurements were done from at least three different mice. Both SEM (*smaller bars*) and SD (*larger bars*) are shown for each measured value. *C*, representative macroscopic currents in individual intercalated cells in response to voltage steps from −90 to +60 from the holding potential of −60 mV in the control (*black*) and following treatment with Ang II (500 nM) for 3 min (*gray*). A micrograph of a typical isolated collecting duct shown on top. The expression of ClC-K2, a marker of intercalated cells (highlighted with *white arrows*), is shown with pseudocolor *red*. Nuclear marker DAPI is shown with pseudocolor *blue*. The scale bar represents 70 μM. *D*, current–voltage relations of the basolateral Cl^−^-selective conductance obtained from voltage step protocols as shown in A in the control (*black*) and upon treatment with Ang II (*gray*). The number of individual recordings is shown. Measurements were done from at least three different mice. Both SEM (*smaller bars*) and SD (*larger bars*) are shown for each measured value. ∗ - significant change (*p* < 0.05) *versus* respective control (one-way ANOVA). DAPI, 4',6-diamidino-2-phenylindole.

The renal collecting duct is a heterogeneous nephron segment containing electrically uncoupled principal and intercalated cells exhibiting different morphology and physiological functions (6, 7). We first used patch clamp electrophysiology in freshly isolated collecting duct to assess Ang II actions on the basolateral conductance in principal and intercalated cells. Since electrical conductance of the apical membrane is much lower than the conductance of the basolateral membrane for both cell types (29, 44), the changes in macroscopic whole cell current chiefly reflect alterations in the electrical conductance of the basolateral membrane. Figure 1*A* shows representative macroscopic currents from aquaporin type 2 (AQP2)-positive principal cells of freshly isolated collecting ducts before and after application of Ang II (500 nM for 3 min). The respective current–voltage relations demonstrate notable inward rectification and reversal around −70 mV (Fig. 1*B*), which is characteristic of the K^+^-selective conductance *via* K_ir_4.1/5.1 channel, as we and others have reported previously (29, 31). However, we did not observe any significant changes in the amplitude of the K_ir_4.1/5.1-mediated K^+^ current in principal cells after treatment with Ang II (Fig. 1, *A* and *B*).

The AQP2-negative intercalated cells exhibited anion-selective conductance with a reversal around −20 mV (Fig. 1, *C* and *D*), which is mediated by the ClC-K2 Cl^−^ channel on the basolateral membrane (45). Of importance, application of Ang II (500 nM for 3 min) significantly increased the amplitude of the Cl^−^-dependent current by almost 2-fold. These results show that Ang II increases the basolateral conductance specifically in the intercalated cells of the collecting duct.

**Figure 2:**
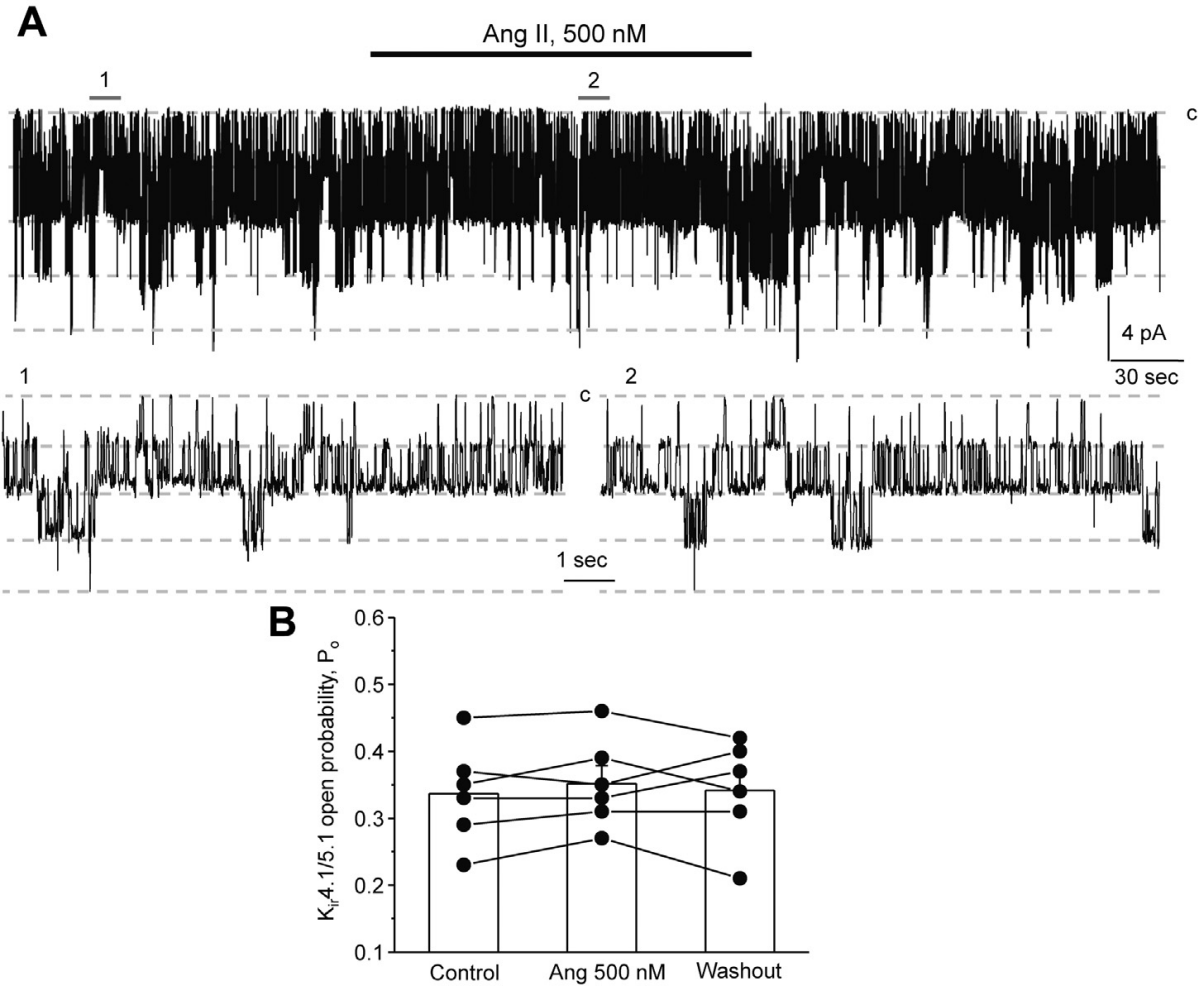
**Ang II does not affect the activity of the basolateral K**_**ir**_**4.1/5.1 channel in principal cells.***A*, representative continuous current trace from a cell-attached patch monitoring activity of the basolateral 40 pS K_ir_4.1/5.1 potassium channels in a principal cell in a freshly isolated collecting duct at the baseline, upon application of 500 nM Ang II (shown with a *line on top*) and following washout with control medium. The patch was clamped to −V_p_ = −40 mV. Areas (1, control) and (2, Ang II) are shown below at an expanded timescale; “c” denotes closed nonconducting state. *B*, summary graph of changes in K_ir_4.1/5.1 open probability (*P*_*o*_) upon treatment with Ang II from paired patch clamp experiments similar to that shown in (*A*). Collecting ducts from at least three different mice were used.

**Figure 3:**
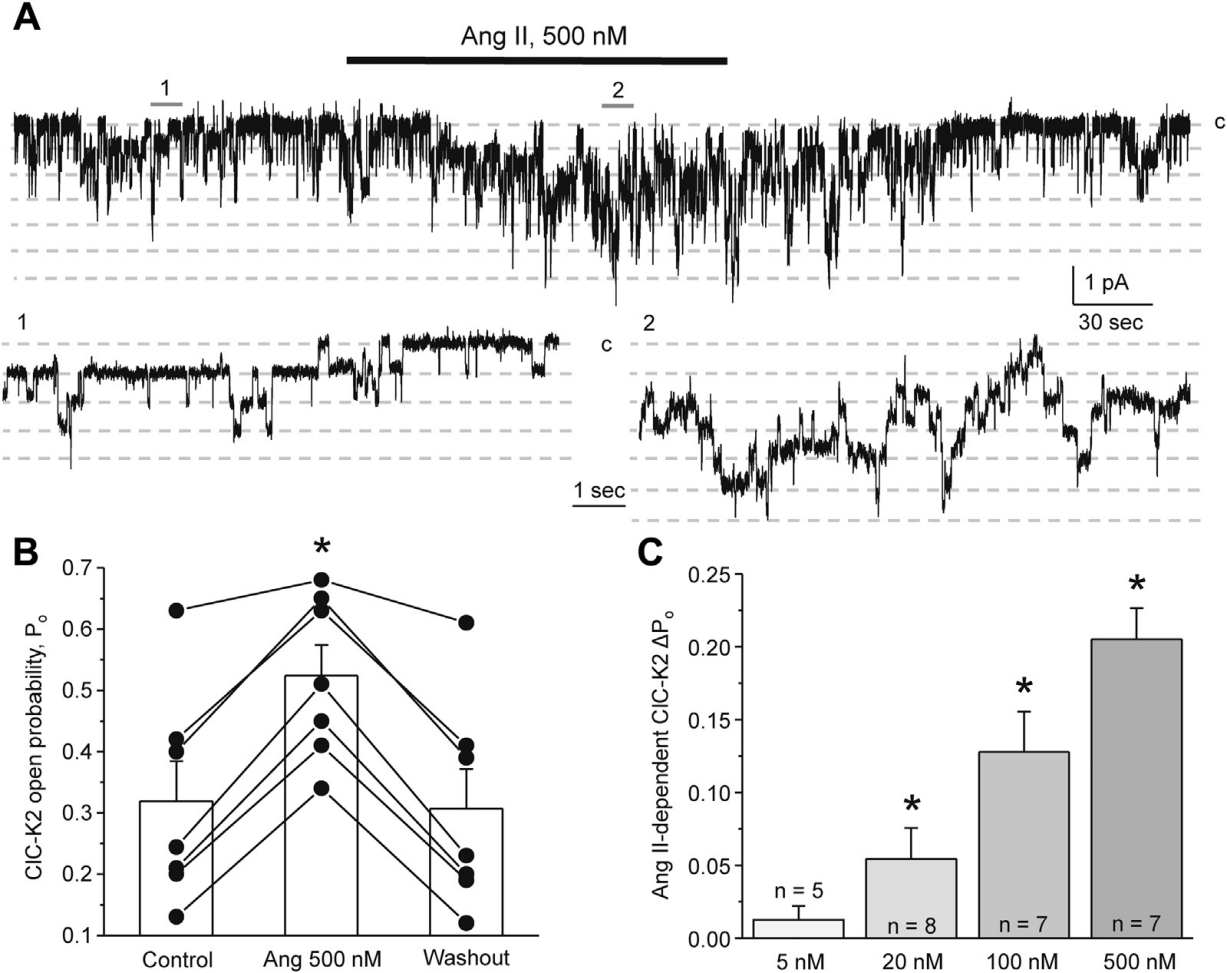
**Ang II stimulates activity of ClC-K2 channel in a dose-dependent manner.***A*, representative continuous current trace from a cell-attached patch monitoring activity of basolateral 10 pS ClC-K2 chloride channels in an intercalated cell in a freshly isolated collecting duct in the control, upon application of 500 nM Ang II (shown with a *line on top*) and following washout with control medium. The patch was clamped to −V_p_ = −60 mV; “c” denotes closed nonconducting state. Areas (1, control) and (2, Ang II) are shown below at an expanded timescale. *B*, summary graph of changes in ClC-K2 open probability (*P*_*o*_) upon treatment with 500 nM Ang II from paired patch clamp experiments similar to that shown in (*A*). *C*, summary graph of average changes in ClC-K2 *P*_*o*_ in individual cells upon application of different Ang II concentrations. ∗ - significant increase (*p* < 0.05) *versus* control (one-way ANOVA). Collecting ducts from at least three different mice were used for each set of experiments.

We next assessed the effects of Ang II on the basolateral conductance of collecting duct cells at the single channel level. As shown in the representative experiment (Fig. 2*A*) and the summary graph (Fig. 2*B*), application of Ang II (500 nM) did not affect the open probability of the 40 pS K_ir_4.1/5.1 channel, the dominant K^+^ channel in the basolateral membrane of the principal cells (29, 31). In contrast, Ang II significantly increased the open probability of the 10 pS ClC-K2 channel (46) in a reversible manner in intercalated cells (Fig. 3*A*). As summarized in Figure 3*B*, the mean open probability was 0.32 ± 0.06, 0.52 ± 0.05, and 0.31 ± 0.06 in the control, after Ang II application, and following washout with control medium, respectively. Ang II increased the ClC-K2 open probability in a dose-dependent manner. As shown in Figure 3*C*, Ang II concentrations higher than 5 nM exhibited a significant stimulatory effect on single channel ClC-K2 activity. It is worth mentioning that similar levels of interstitial Ang II were reported in the kidney (18) arguing for the physiological relevance of Ang II actions on ClC-K2–dependent Cl^−^ conductance in the intercalated cells.

### Ang II acts on AT_1_ receptor to regulate ClC-K2 activity and expression in the collecting duct

**Figure 4:**
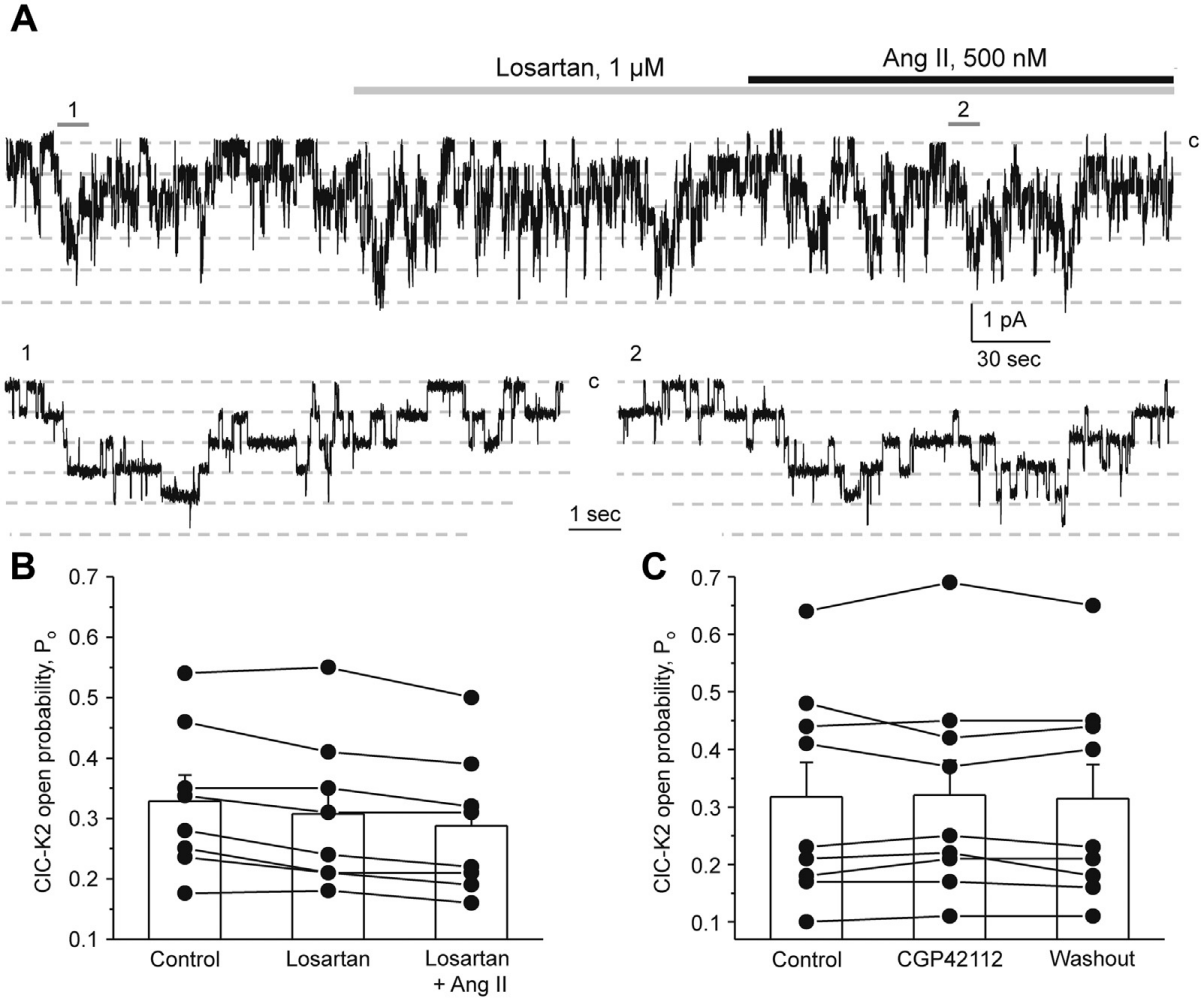
**Ang II increases ClC-K2 activity in intercalated cells by acting on AT**_**1**_**receptors.***A*, representative continuous current trace from a cell-attached patch monitoring activity of basolateral ClC-K2 chloride channels in an intercalated cell of a freshly isolated collecting duct in the control, upon treatment with AT_1_ receptor blocker losartan (1 μM, *gray line*), and Ang II (500 nM, *black line*) in the continued presence of the antagonist. The patch was clamped to −V_p_ = −60 mV; “c” denotes closed nonconducting state. Areas (1, control) and (2, Ang II + losartan) are shown below at an expanded timescale. *B*, summary graph of changes in ClC-K2 open probability (*P*_*o*_) upon treatment with losartan and following Ang II in paired patch clamp experiments similar to that shown in (*A*). *C*, summary graph of changes in ClC-K2 *P*_*o*_ in the control, during treatment with AT_2_ receptor agonist, CGP42112 (100 nM for 3 min), and following washout with control medium. Collecting ducts from at least three different mice were used for each set of experiments.

Expression of both AT_1_ and AT_2_ receptors was reported in the collecting duct cells (25, 26, 27). Thus, we next tested which receptor types are instrumental in transducing stimulatory Ang II actions on ClC-K2. Pretreatment with AT_1_R blocker, losartan (1 μM for 3 min), had no effect on basal ClC-K2 open probability but precluded activation of the channel by Ang II (Fig. 4*A*). As summarized in Figure 4*B*, the mean open probability was 0.32 ± 0.04, 0.31 ± 0.04, and 0.29 ± 0.04 in the control, after pretreatment with losartan, and following Ang II in the presence of losartan, respectively. Stimulation of AT_2_ receptors with a selective agonist, CGP42112 (100 nM for 3 min), did not change the ClC-K2 open probability being 0.31 ± 0.06 and 0.32 ± 0.06 in the control and after the agonist, respectively (Fig. 4*C*). Moreover, stimulation of Mas receptors with Ang 1-7 also had no effect on ClC-K2 activity (Fig. S2). The mean *P*_*o*_ was 0.34 ± 0.05, 0.34 ± 0.05, and 0.33 ± 0.05 in the control, following application of Ang 1-7 (500 nM for 3 min), and washout with control medium, respectively. Altogether, the results in Figure 4 strongly suggest the dominant role for AT_1_R receptor in ClC-K2 activation by Ang II in intercalated cells.

**Figure 5:**
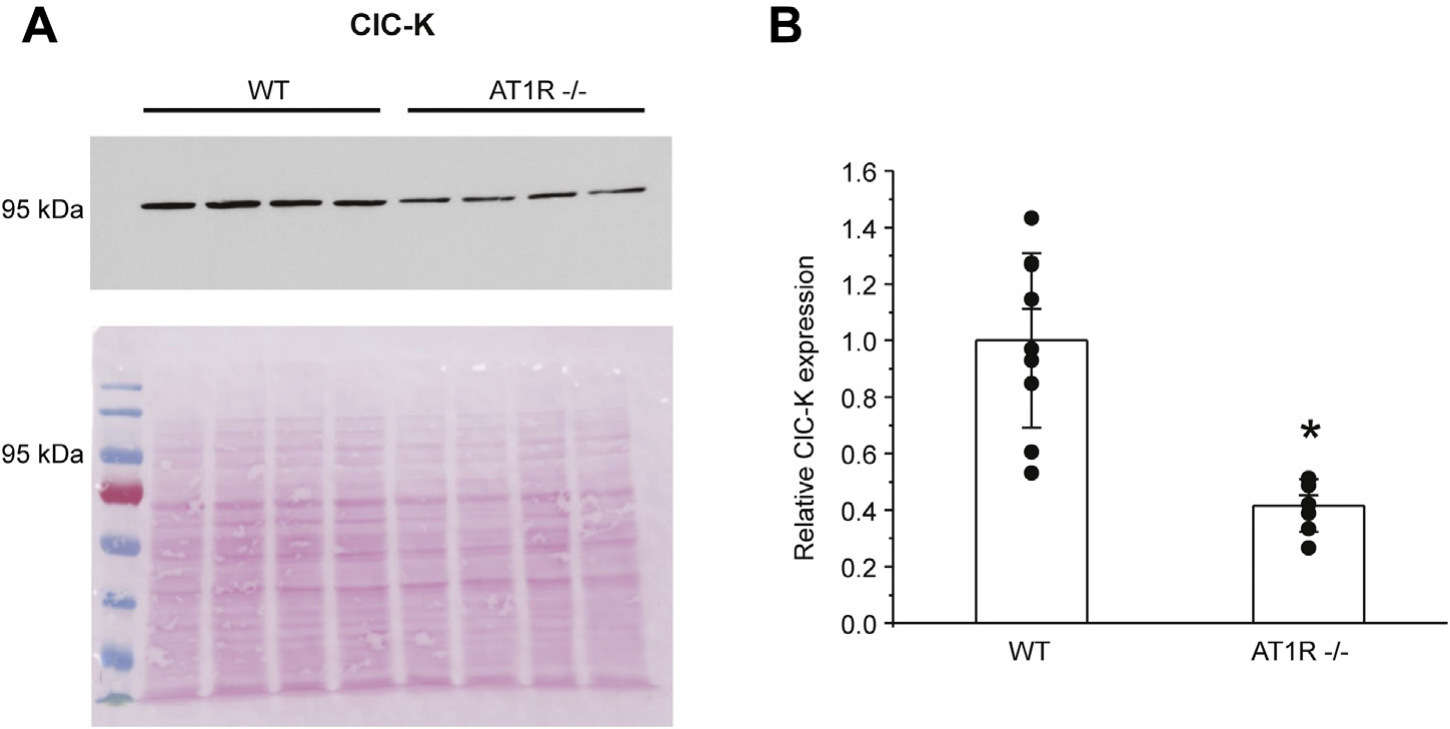
**Deletion of AT**_**1**_**receptors decreases renal ClC-K2 expression.***A*, representative Western blot from whole kidney lysates of WT and AT_1_R −/− mice probed with anti-ClC-K antibodies. The Ponceau red staining of the same nitrocellulose membrane demonstrating equal protein loading is shown in the *bottom panel*. *B*, summary graph comparing ClC-K expression levels in WT and AT_1_R −/− mice. The intensity values were normalized to the total signal of the respective lines in Ponceau red staining. The number of individual mice for each experimental condition is shown. Both SEM (*smaller bars*) and SD (*larger bars*) are shown. ∗ - significant decrease (*p* < 0.05) *versus* control (one-way ANOVA).

We further tested the significance of AT_1_R on renal ClC-K2 expression in mice with genetic deletion of this receptor type. As shown on the representative Western blot in Figure 5*A* and summarized in Figure 5*B*, the intensity of the ClC-K–reporting signal was nearly halved in kidney homogenates from AT_1_R −/− compared with that of WT mice. However, it has to be mentioned that the ClC-K antibodies recognize both ClC-K1 (expressed in the thin ascending limb (47)) and ClC-K2 (expressed in the distal nephron segments (43)). Regardless, the results in Figure 5 indicate that AT_1_R at least partially controls ClC-K2 expression in the kidney.

### Ang II increases ClC-K2 activity in the intercalated cells by activating NOX signaling cascade and generating reactive oxygen species

**Figure 6:**
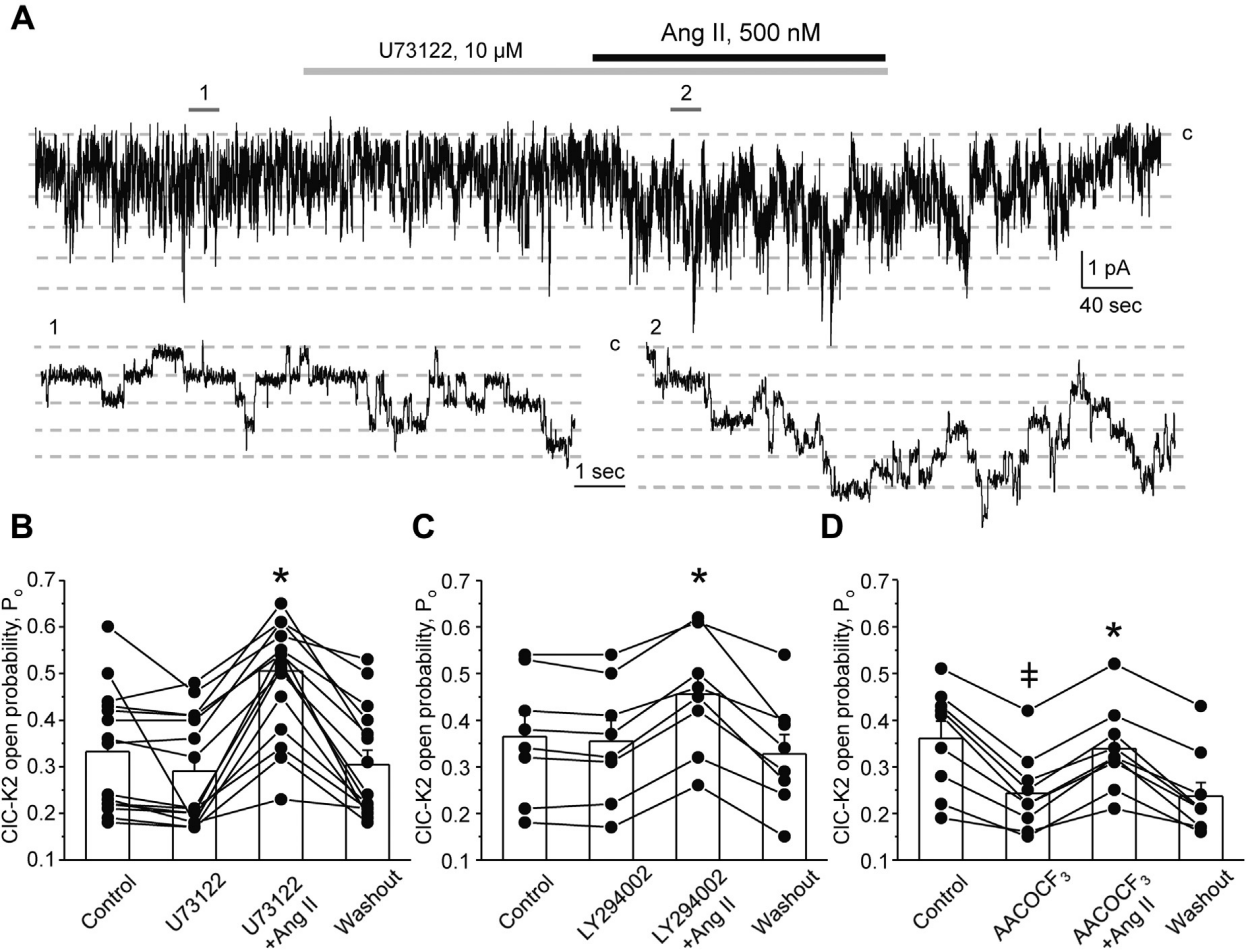
**Inhibition of PLC, PI3-K, and phospholipase A2 signaling cascades does not affect regulation of ClC-K2 activity by Ang II.***A*, representative continuous current trace from a cell-attached patch monitoring activity of basolateral ClC-K2 chloride channels in an intercalated cell of a freshly isolated collecting duct in the control, upon treatment with PLC inhibitor, U73122 (10 μM, *gray* line), Angiotensin II (500 nM, *black line*) in the continued presence of the blocker, and following washout with the control medium. The patch was clamped to −V_p_ = −60 mV; “c” denotes closed nonconducting state. Areas (1, control) and (2, Ang II + U73122) are shown below at an expanded timescale. Summary graphs of changes in ClC-K2 open probability (*P*_*o*_) upon pretreatment with PLC blocker, 10 μM U73122 (*B*), PI3-K blocker, 20 μM LY294002 (*C*), phospholipase A2 blocker, 30 μM AACOCF_3_ (*D*); Ang II application in the continued presence of the respective antagonist, and following washout with control medium, as similarly shown in paired patch clamp experiment in (*A*). ∗ - significant increase (*p* < 0.05) *versus* pretreatment with respective blocker (one-way ANOVA); ^‡^ - significant decrease (*p* < 0.05) *versus* control (one-way ANOVA). Collecting ducts from at least three different mice were used for each set of experiments. PLC, phospholipase C.

Activation of AT_1_R by Ang II can stimulate a variety of intracellular cascades in renal epithelial cells (48). Thus, we next aimed to determine the downstream effector of AT_1_R in mediating ClC-K2 activation in intercalated cells of the collecting duct. As demonstrated by the representative patch clamp experiment in Figure 6*A*, inhibition of the G_q/11_–phospholipase C pathway with U73122 (10 μM) did not significantly alter single channel ClC-K2 activity and did not prevent stimulatory actions of Ang II (500 nM). As summarized in Figure 6*B*, the mean *P*_*o*_ was 0.33 ± 0.03, 0.29 ± 0.03, 0.51 ± 0.04, and 0.31 ± 0.03 in the control, upon pretreatment with U73122 for 3 min, after application of Ang II in the continued presence of the blocker, and following washout with control medium, respectively. Activation of AT_1_R can stimulate phosphoinositide 3 kinase (PI3-K), which is capable of acutely increasing ClC-K2 activity (46). However, pretreatment with PI3-K inhibitor LY294002 (20 μM) for 3 min did not prevent the upregulation of ClC-K2 by Ang II (Fig. 6*C*). Furthermore, inhibition of phospholipase A2, another potential downstream effector of AT_1_R, with AACOCF_3_ (30 μM) significantly inhibited basal ClC-K2 *P*_*o*_ from 0.36 ± 0.03 to 0.24 ± 0.03 but did not abolish Ang II-induced increases in ClC-K2 *P*_*o*_ to 0.33 ± 0.03 (Fig. 6*D*). Overall, we concluded that G_q/11_–phospholipase C, PI3-K, and phospholipase A2 do not play a significant role in the stimulation of ClC-K2 activity by Ang II in intercalated cells of the collecting duct.

**Figure 7:**
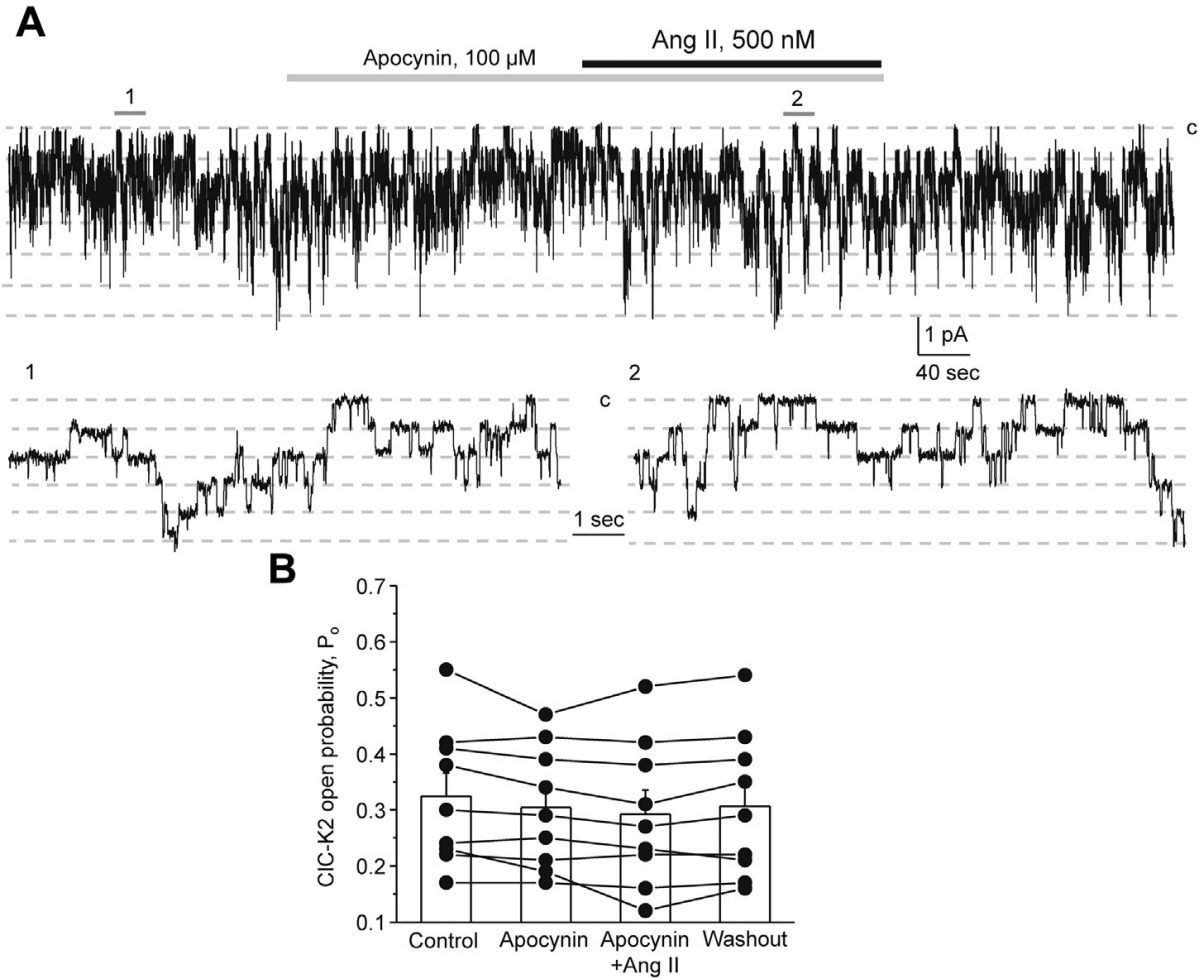
**Ang II increases ClC-K2 activity in intercalated cells in a NOX-dependent manner.***A*, representative continuous current trace from a cell-attached patch monitoring activity of basolateral ClC-K2 chloride channels in an intercalated cell of a freshly isolated collecting duct in the control, upon treatment with NOX inhibitor, apocynin (100 μM, *gray* line), Angiotensin II (500 nM, *black line*) in the continued presence of the blocker, and following washout with the control medium. The patch was clamped to −V_p_ = −60 mV; “c” denotes closed nonconducting state. Areas (1, control) and (2, Ang II + apocynin) are shown below at an expanded timescale. *B*, summary graph of changes in ClC-K2 open probability (*P*_*o*_) upon treatment with apocynin, Ang II in the continued presence of the blocker, and following washout with control medium in paired patch clamp experiments similar to that shown in (*A*). Collecting ducts from at least three different mice were used.

Abundant published evidence demonstrates a marked increase in reactive oxygen species (ROS) levels in renal tubule cells treated with Ang II (49). Thus, we next quantified the action of Ang II on ClC-K2 upon pretreatment with NADPH oxidase (NOX) inhibitor apocynin (100 μM). Apocynin did not affect ClC-K2 basal activity but precluded stimulatory actions of Ang II on the channel (Fig. 7*A*). As summarized in Figure 7*B*, the mean P_o_ was 0.32 ± 0.04, 0.30 ± 0.04, 0.29 ± 0.04, and 0.31 ± 0.04 in the control, upon pretreatment with apocynin for 3 min, after application of Ang II in the continued presence of the NOX blocker, and following washout with control medium, respectively. These results support the view that Ang II increases ClC-K2 activity in a NOX-dependent manner.

**Figure 8:**
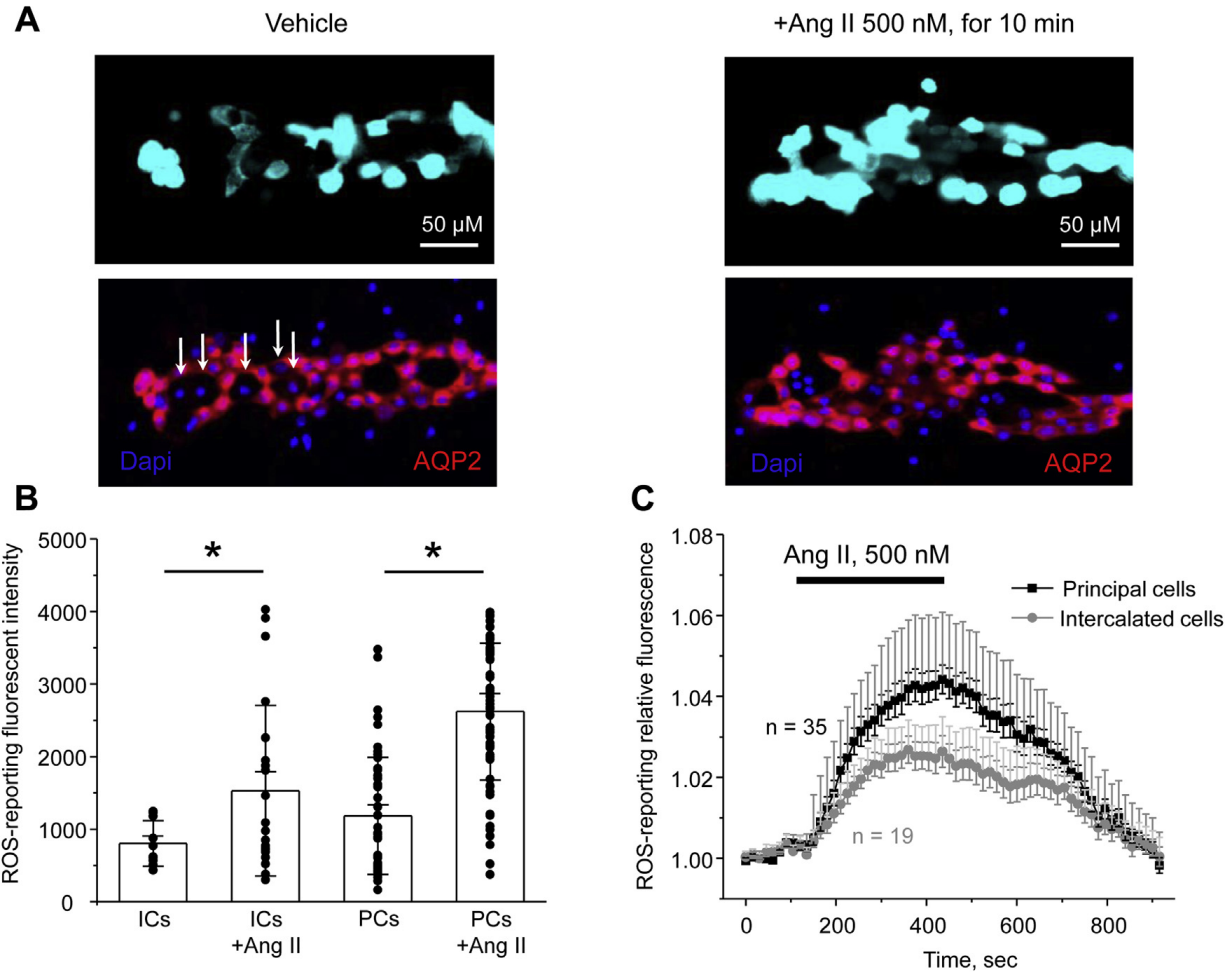
**Ang II increases ROS production in principal and intercalated cells of the collecting duct.***A*, representative micrographs of split-opened collecting ducts loaded with the oxidative stress detection reagent to report ROS levels upon treatment with vehicle (*left*) and Ang II (500 nM) for 10 min (*right*). All images were captured with identical intensity and exposure settings. Confocal micrographs of the same split-opened collectings probed with anti-AQP2 (pseudocolor *red*) are shown below. Examples of AQP2-negative intercalated cells are shown with *white arrows*. Nuclear DAPI staining is shown in pseudocolor *blue*. *B*, summary graph of intensities of ROS-reporting fluorescent signals in individual principal (PCs) and intercalated (ICs) cells in the absence and presence of Ang II treatment, as shown in (*A*). Both SEM (*smaller bars*) and SD (*larger bars*) are shown for each tested group. ∗ - significant increase (*p* < 0.05) *versus* treatment with vehicle as shown with respective *lines on top* (one-way ANOVA). *C*, summary graph comparing the time courses of relative ROS levels in individual principal and intercalated cells upon application of Ang II (500 nM) as shown with the *line on top*. Fluorescent intensities of each cells were normalized to their respective initial values. The number of individual experiments is shown. Collecting ducts from at least three different mice were used. Both SEM (*smaller bars*) and SD (*larger bars*) are shown for each measured time point. DAPI, 4',6-diamidino-2-phenylindole.

We next monitored generation of ROS in response to Ang II in freshly isolated split-opened collecting ducts using fluorescence microscopy. As shown in Figure 8*A*, the overall intensity of the ROS-reporting signal was markedly increased after pretreatment with Ang II (500 nM) for 15 min. We further stained the tested collecting ducts with AQP2 to quantify Ang II-induced ROS generation in principal (AQP2-positive) and intercalated (AQP2-negative, shown with white arrows) cells. As summarized in Figure 8*B*, principal cells exhibited larger ROS levels than intercalated cells at the baseline. Pretreatment with Ang II significantly increased intensities of the ROS-reporting fluorescent signal in both cell types, with the stimulatory effect being moderately more pronounced in principal cells. Finally, we explored the time course of ROS generation in principal and intercalated cells in response to acute administration of Ang II (500 nM). As shown in Figure 8*C*, we detected a rapid and reversible increase in the magnitude of ROS-reporting fluorescent signal in both cell types within 5 min of Ang II application. Consistently with the results in Figure 8*B*, the overall effect was moderately larger in principal cells. Overall, the results in Figure 8 demonstrate that Ang II is capable of increasing ROS production in freshly isolated collecting ducts. Moreover, the time course of Ang II-induced ROS generation (Fig. 8*C*) closely follows the time course of ClC-K2 activation by Ang II (Fig. 3*A*) strongly implying that Ang II increases ClC-K2 *P*_*o*_ in a ROS-dependent manner.

## Discussion

**Figure 9:**
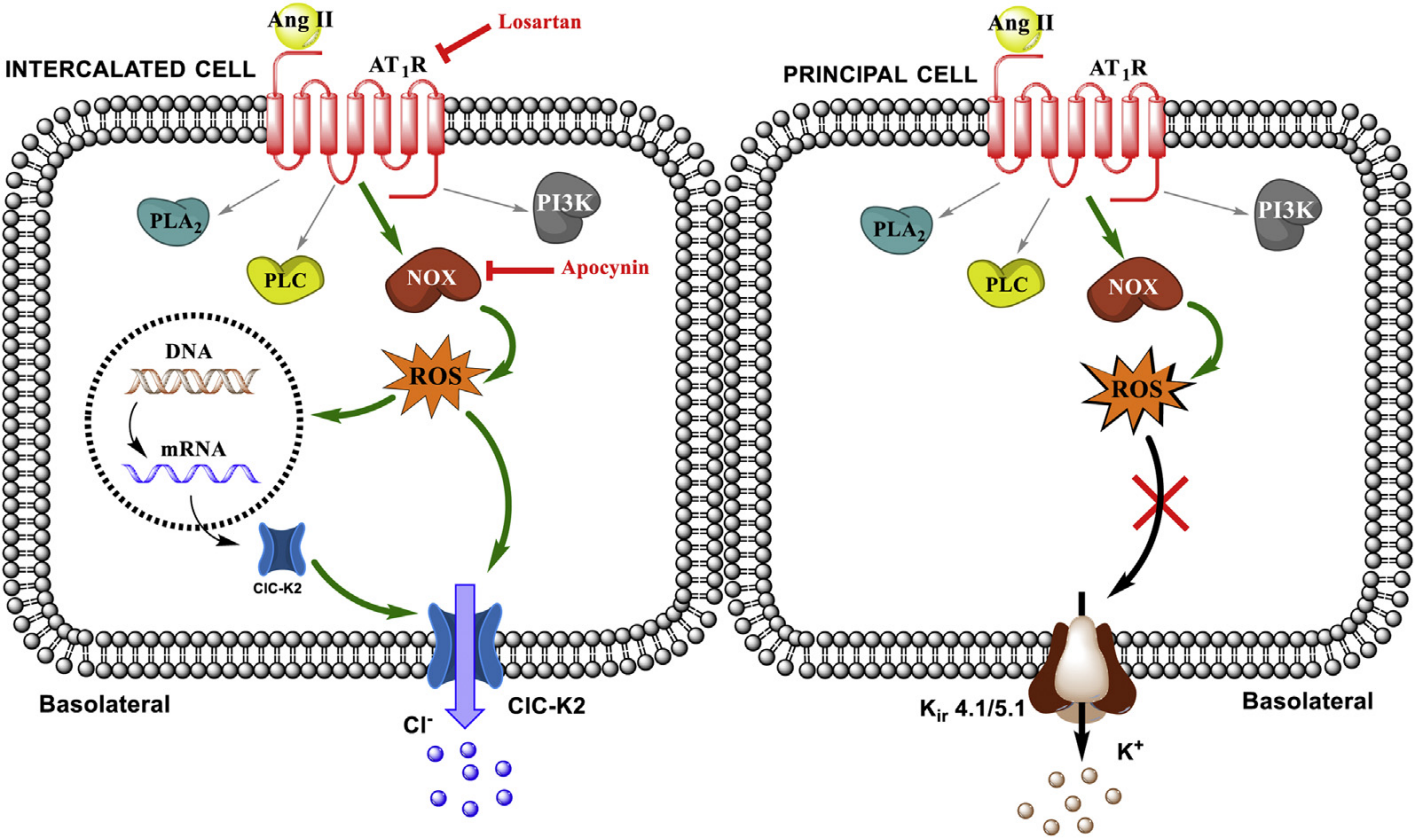
**Principal scheme of Ang II actions on the basolateral conductance in intercalated and principal cells of the collecting duct.***Green arrows* represent stimulatory actions and *red lines* demonstrate successful interruption of the stimulatory pathway with pharmacology. AT_1_R, angiotensin receptor type 1; PLA_2_, phospholipase A2; PLC, phospholipase C; NOX, NADPH oxidase; PI3K, phosphoinositide 3 kinase; ROS, reactive oxygen species.

In the current study, we explored the direct effects of Ang II on the basolateral conductance in principal and intercalated cells of the collecting duct (Fig. 9). We show that Ang II acts on AT_1_R to activate NOX and trigger subsequent ROS generation in both cell types. This pathway stimulates the basolateral Cl^−^ conductance and ClC-K2 activity in intercalated cells but does not alter K^+^-selective conductance and K_ir_4.1/5.1 activity in principal cells despite a moderately greater Ang II-induced ROS generation in this cell type.

Activation of the basolateral Cl^−^ conductance (Fig. 1) and the single channel ClC-K2 activity (Fig. 3) by Ang II provides a strong support to the idea that Ang II stimulates transcellular Cl^−^ reabsorption by the collecting duct intercalated cells. This is in line with the previously reported upregulation of the apical Cl^−^/HCO_3_^−^ exchanger, pendrin, by Ang II in intercalated cells (10). Although it is commonly believed that appreciable Cl^−^ movement occurs in a paracellular manner in the collecting duct (∼30% for rabbits (50)) secondary to the electrogenic Na^+^ reabsorption *via* ENaC by the principal cells, the tight junctions are only marginally more selective for Cl^−^ *versus* Na^+^ (ratio is 1.2–1.3:1) (51) indicating rather passive concomitant NaCl flux, as it similarly occurs in upstream nephron segments (52). Furthermore, blockade of ENaC with amiloride did not abolish lumen-to-bath Cl^−^ movement in perfused collecting ducts (10). We recently showed that ClC-K2 activity in the intercalated cells is inversely related to dietary Cl^−^ intake but not to aldosterone (45). Thus, it is plausible to propose that elevations of Ang II during volume depletion (which is also chloride depletion) increases ENaC-mediated sodium and pendrin/ClC-K2–dependent Cl^−^ reabsorption by acting on principal and intercalated collecting duct cells, respectively (10, 53). Furthermore, Ang II seems to be also critical to determine ClC-K2 expression. Indeed, we found markedly lower ClC-K2 levels in the kidney in mice lacking AT_1_R (Fig. 5). Future studies are necessary to carefully determine the role of Ang II in the regulation of ClC-K2 activity and expression during variations in dietary salt intake.

It is generally believed that the basolateral K_ir_4.1/5.1 channel plays a critical role in setting the resting membrane potential of the basolateral membrane in the collecting duct and upstream segments, such as distal convoluted tubule (32, 54). This, in turn, determines the transepithelial voltage to control NaCl reabsorption. Ang II augments chloride reabsorption in the collecting duct by stimulating apical Cl^−^/HCO_3_^−^ exchange (10) and basolateral Cl^−^ exit *via* ClC-K2 (Figs. 1 and 3) in intercalated cells and increases the apical ENaC-mediated sodium entry in principal cells (14, 53). In this regard, the lack of stimulatory effects of Ang II on K_ir_4.1/5.1 (Figs. 1 and 2) requires a comment. It has been determined that conductance of the basolateral membrane is approximately 10 times larger than conductance of the apical membrane in principal cells (29, 44) with ENaC activity being a rate-limiting step in determining the rate of Na^+^ reabsorption (6). This means that the tandem of Na^+^/K^+^ ATPase and K_ir_4.1/5.1 has more than enough capacity to perform basolateral Na^+^ exit even in the presence of increased ENaC-dependent Na^+^ entry in response to Ang II. Thus, potential stimulation of K_ir_4.1/5.1 activity by Ang II would not further augment Na^+^ reabsorption by the principal cells. On the other side, hyperpolarization of the basolateral membrane (due to augmented K_ir_4.1/5.1 activity) sets up a favorable driving force for the apical K^+^ secretion *via* ROMK (K_ir_1.1) channel (55). Indeed, we recently found that elevated dietary K^+^ intake increases K_ir_4.1/5.1 activity in the collecting duct to facilitate urinary K^+^ excretion (45). In turn, apical K^+^ secretion by the principal cells decreases the electrical driving force for Cl^−^ reabsorption by intercalated cells. Of interest, Ang II has been shown to inhibit ROMK activity (56), which would aid coordination of Na^+^ and Cl^−^ reabsorption by principal and intercalated cells, respectively, in the absence of augmented K^+^ secretion. Overall, it is reasonable to propose that such architecture allows adaptation of the collecting duct cells to different physiological stimuli by switching from Na^+^/K^+^ exchange during hyperkalemia to predominantly NaCl reabsorption during hypovolemia.

Our results suggest a critical role of AT_1_ receptors in mediating stimulatory signal of Ang II to ClC-K2 (Fig. 4). Pretreatment with the AT_1_R blocker, losartan, abolished increases in the ClC-K2 open probability in response to Ang II. Furthermore, we detected a 50% reduction of ClC-K expression in the kidney of AT_1_R −/− mice (Fig. 5) suggesting both regulatory and permissive roles of Ang II-AT_1_R cascade for regulation of ClC-K2–dependent chloride reabsorption in the collecting duct. At this stage, we are not able to determine the relative contribution of the apical and basolateral receptors in this regulation. The basolateral membrane was more readily available in patch clamp studies in freshly isolated collecting ducts (see Fig. 1). However, Ang II elicited a rapid increase in ROS levels in intercalated cells within split-opened area of the collecting duct having exposed the apical membrane (Fig. 8). Thus, our results favor the scenario where apically and basolaterally localized AT_1_R produce complementary stimulatory effects on ClC-K2 activity *via* ROS production. Moreover, both intratubular and interstitial Ang II levels in the kidney were shown to be in the nanomolar range (18), which corresponds to the established concentration range of ClC-K2 regulation by Ang II (Fig. 3*C*). This provides a direct support to the notion that this regulation is physiologically relevant. Although expression of AT2 and Mas receptors have been reported in the collecting duct cells with proposed antihypertensive roles by promoting natriuresis and diuresis (25, 26, 27, 57), their stimulation with CGR42112 and Ang 1-7, respectively, did not affect ClC-K2 activity in the intercalated cells (Fig. 4*C* and Fig. S2). It is possible that the vasoprotective branch of the renin–angiotensin system might play a more pronounced role during volume-expanded hypertensive states and diabetic nephropathy (57). Furthermore, animal sex should be also taken into account, since AT2 receptor expression is higher in females (58). Future studies should carefully determine the contribution of AT_1_ receptor–dependent and –independent mechanisms in the regulation of ClC-K2–mediated Cl^−^ reabsorption in the collecting ducts during normotensive and hypertensive states.

We found Ang II-AT_1_R increases ClC-K2 activity in the intercalated cells by stimulating NOX and following ROS production (Figs. 7 and 8). Of importance, our results demonstrate a striking similarity between the time courses of upregulation of the ClC-K2 open probability (Fig. 3) and ROS generation (Fig. 8) in response to Ang II. Although it is common that ROS can induce covalent modification of specific cysteine residues to alter the functional status of the target proteins, this effect is poorly reversible and does not fit with the observed gradual restoration of the basal ClC-K2 activity upon Ang II washout (Figs. 3 and 6). Of interest, it was shown that an increase in superoxide levels significantly increased intracellular pH, whereas increases in peroxide levels led to intracellular acidification (59, 60). Of note, ClC-K2 exhibits a remarkably steep pH dependence, with acidic (<7.0) and alkaline (>7.5) media leading to acute and reversible decreases and increases in channel activity, respectively (46, 61). A potential mechanism likely involves protonation/deprotonation of a histidine residue (H497) of the channel (62). Thus, it is plausible to propose that the Ang II-AT_1_R-NOX pathway promotes generation of superoxide to increase cytosolic pH to activate ClC-K2 in intercalated cells. At the same time, K_ir_4.1/5.1 is also known to be sensitive to pH (31, 63). It is possible that Ang II leads to generation of both superoxide and peroxide in principal cells but mostly superoxide in intercalated cells. This would explain the higher ROS (superoxide and peroxide together) levels shown in Figure 8 but potentially little pH changes and thus lack of K_ir_4.1/5.1 regulation by Ang II in principal cells. We will consider to investigate this intriguing aspect in the future.

Although ROS-dependent redox-signaling processes contribute significantly to the normal cellular responses to Ang II, excessive ROS accumulation drives proinflammatory and profibrotic actions of Ang II contributing to endothelial dysfunction, fibrosis, and the development of hypertension (64). Indeed, we previously showed that Ang II increases ENaC activity by increasing ROS in the collecting duct principal cells (14) and this is an important mechanism for stimulation of ENaC-dependent Na^+^ reabsorption in response to dietary sodium deficiency (53). However, chronic Ang II infusion stimulates ENaC activity far beyond the physiological range independently of aldosterone thus contributing to excessive volume retention and hypertension (13). Cl^−^ is the principal extracellular anion accounting for over 70% of the total negative ion content. Jointly with Na^+^, Cl^−^ is the major contributor to extracellular volume and osmolarity. Although abnormal regulation of Na^+^ balance is considered to be central for the development of elevated blood pressure, accumulated evidence argues that the Cl^−^ component might play an even more important role in the pathology of salt-sensitive hypertension (65). For instance, Dahl salt-sensitive or stroke-prone spontaneously hypertensive rats develop elevated blood pressure when fed with high-NaCl diet but not high Na^+^ bicarbonate or other Cl^−^ substitutes (66, 67, 68). This article demonstrates that physiologically relevant Ang II levels stimulate ClC-K2 activity and by extension transcellular Cl^−^ reabsorption in the collecting duct, the site directly involved in the regulation of urinary electrolyte excretion to match dietary intake and setting salt sensitivity of blood pressure. Overall, we propose that upregulation of ClC-K2 by Ang II is critical for protection of the circulatory volume during hypovolemic states, whereas overactivation of ClC-K2 might contribute to the pathophysiology of Ang II–dependent hypertension.

## Experimental procedures

### Reagents and animals

All chemicals and materials were from Sigma, VWR, and Tocris unless noted otherwise and were of reagent grade. For experiments, C57BL/6J mice (Charles River Laboratories) and B6.129P2-*Agtr1*^*tm1Unc*^/J (AT_1a_R −/−, JAX strain #002682, the dominant AT_1_ receptor isoform in the kidney (69)) 6 to 10 weeks old were used. In order to minimize sex-related variations in the measured experimental parameters, only males were used for experiments. Animal use and welfare adhered to the NIH Guide for the Care and Use of Laboratory Animals following protocols reviewed and approved by the Animal Care and Use Committee of the University of Texas Health Science Center at Houston.

### Tissue isolation

The procedure for isolation of the collecting ducts suitable for electrophysiology followed previously published protocols (45, 70, 71). Briefly, mice were sacrificed by CO_2_ administration followed by cervical dislocation, and the kidneys were removed immediately. Kidneys were cut into thin slices (<1 mm) with slices placed into ice-cold Ringer solution containing (in mM) 150 NaCl, 5 KCl, 1 CaCl_2_, 2 MgCl_2_, 5 glucose, and 10 Hepes (pH 7.35). Straight cortical-to-medullary sectors, containing approximately 30 to 50 renal tubules, were isolated by microdissection using watchmaker forceps under a stereomicroscope. To dissolve the basal lamina and to get direct access to the basolateral membrane, isolated sectors were further incubated in the Ringer solution containing 0.8 mg/ml collagenase type I (Alfa Aesar) and 5 mg/ml of dispase II (Roche Diagnostics) for 20 min at 37 °C followed by extensive washout. Individual collecting ducts were visually identified by their morphological features (pale color; coarse surface) and were mechanically isolated from the sectors by microdissection. The collecting ducts were further verified by positive expression of AQP2 water channel with immunofluorescent microscopy, as detailed below. Isolated collecting ducts were attached to a 5 x 5 mm cover glass coated with poly-L-lysine. A cover glass containing a collecting duct was placed in a chamber mounted on an inverted Nikon Eclipse Ti microscope and perfused with the Ringer solution at room temperature. The samples were used within 1 to 2 h after isolation. For each experimental condition, collecting ducts from at least three different mice were analyzed.

### Whole cell currents and membrane potential in isolated collecting ducts

Whole cell currents in collecting duct cells were measured under voltage-clamp conditions in the perforated-patch mode with gigaohm seals formed on the basolateral membrane, as described (45, 70). Patch clamp recordings were acquired with an Axopatch 200B (Molecular Devices) patch clamp amplifier interfaced *via* a Digidata 1440 (Molecular Devices) to a computer running the pClamp 10.7 (Molecular Devices). The bath solution was (in mM): 150 NaCl, 5 KCl, 1 CaCl_2_, 2 MgCl_2_, 5 glucose, and 10 Hepes (pH 7.35). Freshly made amphotericin-B, 400 μM (Enzo Life Sciences) was dissolved in the pipette solution containing (in mM):150 KAcetate, 5 KCl, 2 MgCl_2_, and 10 Hepes (pH 7.35) by ultrasonication. Recording pipettes had resistances of 3 to 5 MΩ. Electrical recordings were made once the access resistance from the pipette to the cell interior reduced to less than 15 MΩ, usually 5 to 10 min after achieving a pipette-to-membrane seal resistance of 5 to 10 GΩ. The capacity of individual cells (∼15 pF) was manually compensated. Principal and intercalated cells were further distinguished by their electrical properties with principal cells having K^+^-selective cation conductance and a highly negative resting membrane potential around −70 mV, whereas intercalated cells have Cl^−^-selective anion conductance and resting membrane potential around −20 mV, as we demonstrated previously (29).

### Single channel recordings in isolated collecting ducts

The activity of K_ir_4.1/5.1 and ClC-K2 channels in freshly isolated collecting ducts was determined in cell-attached patches on the basolateral membrane of principal and intercalated cells, respectively, under voltage-clamp conditions, as previously described (45, 70). Recording pipettes had resistances of 8 to 10 MΩ. Bath and pipette solutions were (in mM): 150 NaCl, 5 KCl, 1 CaCl_2_, 2 MgCl_2_, 5 glucose, and 10 Hepes (pH 7.35); and 150 KCl, 2 MgCl_2_, and 10 Hepes (pH 7.35). In the cell attached configuration, the actual voltage applied to a membrane patch (V_patch_) is a sum of the pipette voltage and the resting basolateral membrane potential of principal (V_basolateral_, which is close to −70 mV for principal and −20 mV for intercalated cells, see Fig. 1, *B* and *D*). Currents were low-pass filtered at 1 kHz with an eight-pole Bessel filter (Warner Instruments). Events were inspected visually prior to acceptance. Channel activity (*NP*_o_) and open probability (*P*_o_) were assessed using Clampfit 10.7 (Molecular Devices). Channel activity in individual patches, defined as *NP*_o_, was calculated using the following equation: *NP*_o_ = (*t*_1_ + 2*t*_2_ + …+*nt*_*n*_), where *N* is the number of active channels (K_ir_4.1/5.1 or ClC-K2) in a patch and *t*_*n*_ is the fractional open time spent at each of the observed current levels. *P*_o_ was calculated by dividing *NP*_o_ by the maximal number of simultaneously active channels within a patch (*N*) as defined by all-point amplitude histograms. For representation, current traces were filtered at 200 Hz and corrected for a slow baseline drift as necessary.

### Western blotting

Immediately after dissection kidneys were placed on ice, decapsulated, and homogenized in three volumes of ice-cold lysis buffer containing 50 mM TrisCl, 5 mM EDTA and 1% Triton X-100 (pH 7.5) supplemented with Complete Mini protease and PhosSTOP phosphatase inhibitor cocktails (Roche Diagnostics). The homogenates were centrifuged at 1000*g* for 15 min at +4 °C, and the sediment was discarded. Protein concentration was determined with a Bradford assay using bovine serum albumin as a standard. The samples (40 μg/lane) were separated on 9% polyacrylamide gels at 150 V for 90 min and transferred to a nitrocellulose membrane for 70 min at 100 V. Equal protein load was verified by Ponceau red staining using standard procedures. Nitrocellulose membranes were incubated with primary anti-ClC-K antibodies (rabbit polyclonal, 1:1000 Alomone Labs, Cat. # ACL-004) overnight at +4 °C. Upon washout (three times for 10 min in TBS-Tween), the membrane was incubated with peroxidase-conjugated goat anti-rabbit (1:10,000, Jackson ImmunoResearch Laboratories) secondary antibodies for 1 h at room temperature. Blots were quantified using ImageJ 1.50e software (NIH). The intensities of the studied protein bands were normalized to the total signal of the respective line in Ponceau red staining.

### Total ROS detection

Freshly isolated split-opened collecting ducts were loaded with the oxidative stress detection reagent (Enzo Life Sciences, ENZ-51011) in 1X Wash Buffer for 45 min at room temperature according to the manufacturer’s protocol. Fluorescent images were recorded with the same exposure time (4 ms) with a Nikon Ti-S Wide-Field Fluorescence Imaging System (Nikon Instruments) integrated with Lambda XL light source (Sutter Instrument) and QIClick 1.4 megapixel monochrome CCD camera (QImaging) *via* NIS Elements 4.3 Imaging Software (Nikon Instruments). The collecting ducts were imaged with Nikon fluorescence microscope with excitation at 490 nm and emission at 525 nm using a 40X Nikon Super Fluor objective, and regions of interest were drawn for individual cells. The efficiency of the total ROS detection kit was tested on subconfluent mpkCCD_c14_ cells, a generally accepted model of the collecting duct principal cells, as we similarly did previously (14). As shown on the representative micrographs in Fig. S1*A* and summarized in Fig. S1*B*, addition of ROS inducer pyocyanin (200 μM for the last 20 min of incubation) drastically increased the intensity of ROS-reporting fluorescent signal, which was largely precluded by concomitant incubation with the negative control reagent (N-acetyl-L-cysteine, 10 mM) for 30 min. For the experiments, Ang II (500 nM) or vehicle were added to the freshly isolated split-opened collecting ducts for the last 10 min of the incubation with the oxidative stress detection reagent. In another set of experiments, the time course of changes in ROS in response to perfusion of Ang II (500 nM) was assessed by sampling the fluorescent intensities at 525 nm every 15 s.

### Immunofluorescent microscopy

Immediately after ROS measurements, *s*plit-opened collecting ducts were fixed with 10% neutral buffer formalin for 15 min at room temperature. After fixation, the samples were permeabilized by addition of 1% SDS in PBS for 10 min and washed in PBS for 5 min. Nonspecific staining was blocked with 1% BSA in PBS for 1 h at room temperature. The samples were incubated overnight at +4 °C with anti-Aquaporin 2 antibody (1:4000 dilution; Alomone Labs, Cat. # AQP-002). After washing with PBS, the samples were incubated with goat anti-rabbit IgG labeled with Alexa Fluor 594 (1:1000 dilution; Invitrogen) for 1 h at room temperature in the dark. For experiments with double staining, the samples were incubated with anti-Aquaporin 2 antibody (1:4000 dilution; Alomone Labs, Cat # AQP-002) overnight at 4 °C. After washing with PBS, the samples were incubated with goat anti-rabbit IgG labeled with Alexa Fluor 488 (1:2000 dilution; Invitrogen) for 1 h at room temperature in the dark. Subsequently, nonspecific staining was blocked with 10% rabbit serum for 30 min and samples were incubated with anti-CLC-K antibody (1:500 dilution; Alomone, Cat # ACL-004) conjugated with goat anti-rabbit IgG labeled with Alexa Fluor 594 for 2 h at 37 °C in the dark. After washing with PBS (three times for 5 min) the samples were stained with 4',6-diamidino-2-phenylindole (500 nM concentration, Calbiochem) to visualize nuclei. The samples were dehydrated and mounted with Fluoromount-G (SouthernBiotech, Cat# 0100-01). Labeled tubules were examined with an inverted Nikon Eclipse Ti fluorescent microscope using a 40X Plan-Fluor (1.3 NA) objective. Samples were excited with 405- and 561-nm laser diodes, and emission was captured with a 16-bit Cool SNAP HQ^2^ camera (Photometrics) interfaced to a PC running NIS elements software.

### Data analysis

All summarized data are reported as mean ± SEM and ±SD for nonpaired experiments and mean ± SEM for paired patch clamp studies, as indicated in respective figure legends. Statistical comparisons were made using one-way ANOVA with post hoc Tukey test or one-way repeated measures ANOVA with post hoc Bonferroni test (for paired experiments within the same group). *p* Value less than 0.05 was considered significant.

## Data availability

All data from this study are contained within the article including Supplemental Information.

## Conflict of interest

The authors declare that they have no conflicts of interest with the contents of this article.

## References

[1] Reboussin D.M., Allen N.B., Griswold M.E., Guallar E., Hong Y., Lackland D.T., Miller E.P.R., Polonsky T., Thompson-Paul A.M., Vupputuri S. (2018). Systematic review for the 2017 ACC/AHA/AAPA/ABC/ACPM/AGS/APhA/ASH/ASPC/NMA/PCNA guideline for the prevention, detection, evaluation, and management of high blood pressure in adults: A report of the American College of Cardiology/American Heart Association task force on clinical practice guidelines. Circulation.

[2] Kotchen T.A., Cowley A.W., Frohlich E.D. (2013). Salt in health and disease--a delicate balance. N. Engl. J. Med..

[3] Meneton P., Jeunemaitre X., de Wardener H.E., MacGregor G.A. (2005). Links between dietary salt intake, renal salt handling, blood pressure, and cardiovascular diseases. Physiol. Rev..

[4] Pratt J.H. (2005). Central role for ENaC in development of hypertension. J. Am. Soc. Nephrol..

[5] Bhalla V., Hallows K.R. (2008). Mechanisms of ENaC regulation and clinical implications. J. Am. Soc. Nephrol..

[6] Pearce D., Soundararajan R., Trimpert C., Kashlan O.B., Deen P.M., Kohan D.E. (2014). Collecting duct principal cell transport processes and their regulation. Clin. J. Am. Soc. Nephrol..

[7] Roy A., Al-bataineh M.M., Pastor-Soler N.M. (2015). Collecting duct intercalated cell function and regulation. Clin. J. Am. Soc. Nephrol..

[8] Masilamani S., Kim G.H., Mitchell C., Wade J.B., Knepper M.A. (1999). Aldosterone-mediated regulation of ENaC alpha, beta, and gamma subunit proteins in rat kidney. J. Clin. Invest..

[9] Staruschenko A. (2012). Regulation of transport in the connecting tubule and cortical collecting duct. Compr. Physiol..

[10] Pech V., Kim Y.H., Weinstein A.M., Everett L.A., Pham T.D., Wall S.M. (2007). Angiotensin II increases chloride absorption in the cortical collecting duct in mice through a pendrin-dependent mechanism. Am. J. Physiol. Renal Physiol..

[11] Wall S.M., Weinstein A.M. (2013). Cortical distal nephron Cl^-^ transport in volume homeostasis and blood pressure regulation. Am. J. Physiol. Renal Physiol..

[12] Peti-Peterdi J., Warnock D.G., Bell P.D. (2002). Angiotensin II directly stimulates ENaC activity in the cortical collecting duct via AT_1_ receptors. J. Am. Soc. Nephrol..

[13] Mamenko M., Zaika O., Prieto M.C., Jensen V.B., Doris P.A., Navar L.G., Pochynyuk O. (2013). Chronic angiotensin II infusion drives extensive aldosterone-independent epithelial Na^+^ channel activation. Hypertension.

[14] Mamenko M., Zaika O., Ilatovskaya D.V., Staruschenko A., Pochynyuk O. (2012). Angiotensin II increases activity of the epithelial Na^+^ channel (ENaC) in distal nephron additively to aldosterone. J. Biol. Chem..

[15] Gonzalez-Villalobos R.A., Satou R., Ohashi N., Semprun-Prieto L.C., Katsurada A., Kim C., Upchurch G.M., Prieto M.C., Kobori H., Navar L.G. (2010). Intrarenal mouse renin-angiotensin system during ANG II-induced hypertension and ACE inhibition. Am. J. Physiol. Renal Physiol..

[16] Gonzalez-Villalobos R.A., Seth D.M., Satou R., Horton H., Ohashi N., Miyata K., Katsurada A., Tran D.V., Kobori H., Navar L.G. (2008). Intrarenal angiotensin II and angiotensinogen augmentation in chronic angiotensin II-infused mice. Am. J. Physiol. Renal Physiol..

[17] Navar L.G., Prieto M.C., Satou R., Kobori H. (2011). Intrarenal angiotensin II and its contribution to the genesis of chronic hypertension. Curr. Opin. Pharmacol..

[18] Navar L.G., Lewis L., Hymel A., Braam B., Mitchell K.D. (1994). Tubular fluid concentrations and kidney contents of angiotensins I and II in anesthetized rats. J. Am. Soc. Nephrol..

[19] Siragy H.M., Howell N.L., Ragsdale N.V., Carey R.M. (1995). Renal interstitial fluid angiotensin. Modulation by anesthesia, epinephrine, sodium depletion, and renin inhibition. Hypertension.

[20] Zaman M.A., Oparil S., Calhoun D.A. (2002). Drugs targeting the renin-angiotensin-aldosterone system. Nat. Rev. Drug Discov..

[21] Seva P.B., van der L.N., Verdonk K., Roks A.J., Hoorn E.J., Danser A.H. (2012). Key developments in renin-angiotensin-aldosterone system inhibition. Nat. Rev. Nephrol..

[22] Kaschina E., Unger T. (2003). Angiotensin AT1/AT2 receptors: Regulation, signalling and function. Blood Press.

[23] Crowley S.D., Coffman T.M. (2012). Recent advances involving the renin-angiotensin system. Exp. Cell Res..

[24] Berry C., Touyz R., Dominiczak A.F., Webb R.C., Johns D.G. (2001). Angiotensin receptors: Signaling, vascular pathophysiology, and interactions with ceramide. Am. J. Physiol. Heart Circ. Physiol..

[25] Carey R.M., Wang Z.Q., Siragy H.M. (2000). Role of the angiotensin type 2 receptor in the regulation of blood pressure and renal function. Hypertension.

[26] Miyata N., Park F., Li X.F., Cowley A.W. (1999). Distribution of angiotensin AT1 and AT2 receptor subtypes in the rat kidney. Am. J. Physiol..

[27] Ozono R., Wang Z.Q., Moore A.F., Inagami T., Siragy H.M., Carey R.M. (1997). Expression of the subtype 2 angiotensin (AT2) receptor protein in rat kidney. Hypertension.

[28] Ortiz R.M., Graciano M.L., Seth D., Awayda M.S., Navar L.G. (2007). Aldosterone receptor antagonism exacerbates intrarenal angiotensin II augmentation in ANG II-dependent hypertension. Am. J. Physiol. Renal Physiol..

[29] Zaika O., Palygin O., Tomilin V., Mamenko M., Staruschenko A., Pochynyuk O. (2016). Insulin and IGF-1 activate K_ir_4.1/5.1 channels in cortical collecting duct principal cells to control basolateral membrane voltage. Am. J. Physiol. Renal Physiol..

[30] Muto S., Yasoshima K., Yoshitomi K., Imai M., Asano Y. (1990). Electrophysiological identification of alpha- and beta-intercalated cells and their distribution along the rabbit distal nephron segments. J. Clin. Invest..

[31] Lachheb S., Cluzeaud F., Bens M., Genete M., Hibino H., Lourdel S., Kurachi Y., Vandewalle A., Teulon J., Paulais M. (2008). K_ir_4.1/K_ir_5.1 channel forms the major K^+^ channel in the basolateral membrane of mouse renal collecting duct principal cells. Am. J. Physiol. Renal Physiol..

[32] Terker A.S., Zhang C., McCormick J.A., Lazelle R.A., Zhang C., Meermeier N.P., Siler D.A., Park H.J., Fu Y., Cohen D.M., Weinstein A.M., Wang W.H., Yang C.L., Ellison D.H. (2015). Potassium modulates electrolyte balance and blood pressure through effects on distal cell voltage and chloride. Cell Metab..

[33] Cuevas C.A., Su X.T., Wang M.X., Terker A.S., Lin D.H., McCormick J.A., Yang C.L., Ellison D.H., Wang W.H. (2017). Potassium sensing by renal distal tubules requires K_ir_4.1. J. Am. Soc. Nephrol..

[34] Bockenhauer D., Feather S., Stanescu H.C., Bandulik S., Zdebik A.A., Reichold M., Tobin J., Lieberer E., Sterner C., Landoure G., Arora R., Sirimanna T., Thompson D., Cross J.H., van't Hoff W. (2009). Epilepsy, ataxia, sensorineural deafness, tubulopathy, and KCNJ10 mutations. N. Engl. J. Med..

[35] Scholl U.I., Choi M., Liu T., Ramaekers V.T., Hausler M.G., Grimmer J., Tobe S.W., Farhi A., Nelson-Williams C., Lifton R.P. (2009). Seizures, sensorineural deafness, ataxia, mental retardation, and electrolyte imbalance (SeSAME syndrome) caused by mutations in KCNJ10. Proc. Natl. Acad. Sci. U. S. A..

[36] Palygin O., Levchenko V., Ilatovskaya D.V., Pavlov T.S., Pochynyuk O.M., Jacob H.J., Geurts A.M., Hodges M.R., Staruschenko A. (2017). Essential role of K_ir_5.1 channels in renal salt handling and blood pressure control. JCI Insight.

[37] Hennings J.C., Andrini O., Picard N., Paulais M., Huebner A.K., Cayuqueo I.K., Bignon Y., Keck M., Corniere N., Bohm D., Jentsch T.J., Chambrey R., Teulon J., Hubner C.A., Eladari D. (2017). The ClC-K2 chloride channel is critical for salt handling in the distal nephron. J. Am. Soc. Nephrol..

[38] Nissant A., Paulais M., Lachheb S., Lourdel S., Teulon J. (2006). Similar chloride channels in the connecting tubule and cortical collecting duct of the mouse kidney. Am. J. Physiol. Renal Physiol..

[39] Lourdel S., Paulais M., Marvao P., Nissant A., Teulon J. (2003). A chloride channel at the basolateral membrane of the distal-convoluted tubule: A candidate ClC-K channel. J. Gen. Physiol..

[40] Simon D.B., Bindra R.S., Mansfield T.A., Nelson-Williams C., Mendonca E., Stone R., Schurman S., Nayir A., Alpay H., Bakkaloglu A., Rodriguez-Soriano J., Morales J.M., Sanjad S.A., Taylor C.M., Pilz D. (1997). Mutations in the chloride channel gene, CLCNKB, cause Bartter's syndrome type III. Nat. Genet..

[41] Andrini O., Keck M., Briones R., Lourdel S., Vargas-Poussou R., Teulon J. (2015). ClC-K chloride channels: Emerging pathophysiology of Bartter syndrome type 3. Am. J. Physiol. Renal Physiol..

[42] Birkenhager R., Otto E., Schurmann M.J., Vollmer M., Ruf E.M., Maier-Lutz I., Beekmann F., Fekete A., Omran H., Feldmann D., Milford D.V., Jeck N., Konrad M., Landau D., Knoers N.V. (2001). Mutation of BSND causes Bartter syndrome with sensorineural deafness and kidney failure. Nat. Genet..

[43] Kobayashi K., Uchida S., Mizutani S., Sasaki S., Marumo F. (2001). Intrarenal and cellular localization of CLC-K2 protein in the mouse kidney. J. Am. Soc. Nephrol..

[44] Gray D.A., Frindt G., Zhang Y.Y., Palmer L.G. (2005). Basolateral K^+^ conductance in principal cells of rat CCD. Am. J. Physiol. Renal Physiol..

[45] Tomilin V.N., Zaika O., Subramanya A.R., Pochynyuk O. (2018). Dietary K^+^ and Cl^-^ independently regulate basolateral conductance in principal and intercalated cells of the collecting duct. Pflugers Arch..

[46] Zaika O., Mamenko M., Boukelmoune N., Pochynyuk O. (2015). IGF-1 and insulin exert opposite actions on ClC-K2 activity in the cortical collecting ducts. Am. J. Physiol. Renal Physiol..

[47] Matsumura Y., Uchida S., Kondo Y., Miyazaki H., Ko S.B., Hayama A., Morimoto T., Liu W., Arisawa M., Sasaki S., Marumo F. (1999). Overt nephrogenic diabetes insipidus in mice lacking the CLC-K1 chloride channel. Nat. Genet..

[48] Navar L.G., Kobori H., Prieto M.C., Gonzalez-Villalobos R.A. (2011). Intratubular renin-angiotensin system in hypertension. Hypertension.

[49] Lara L.S., McCormack M., Semprum-Prieto L.C., Shenouda S., Majid D.S., Kobori H., Navar L.G., Prieto M.C. (2012). AT1 receptor-mediated augmentation of angiotensinogen, oxidative stress, and inflammation in ANG II-salt hypertension. Am. J. Physiol. Renal Physiol..

[50] Schuster V.L., Stokes J.B. (1987). Chloride transport by the cortical and outer medullary collecting duct. Am. J. Physiol..

[51] Warden D.H., Schuster V.L., Stokes J.B. (1988). Characteristics of the paracellular pathway of rabbit cortical collecting duct. Am. J. Physiol..

[52] Monzon C.M., Garvin J.L. (2015). Nitric oxide decreases the permselectivity of the paracellular pathway in thick ascending limbs. Hypertension.

[53] Mamenko M., Zaika O., Tomilin V., Jensen V.B., Pochynyuk O. (2018). Compromised regulation of the collecting duct ENaC activity in mice lacking AT_1a_ receptor. J. Cell. Physiol..

[54] Zaika O.L., Mamenko M., Palygin O., Boukelmoune N., Staruschenko A., Pochynyuk O. (2013). Direct inhibition of basolateral K_ir_4.1/5.1 and K_ir_4.1 channels in the cortical collecting duct by dopamine. Am. J. Physiol. Renal Physiol..

[55] Muto S., Sansom S., Giebisch G. (1988). Effects of a high potassium diet on electrical properties of cortical collecting ducts from adrenalectomized rabbits. J. Clin. Invest..

[56] Wei Y., Zavilowitz B., Satlin L.M., Wang W.H. (2007). Angiotensin II inhibits the ROMK-like small conductance K channel in renal cortical collecting duct during dietary potassium restriction. J. Biol. Chem..

[57] Dilauro M., Burns K.D. (2009). Angiotensin-(1-7) and its effects in the kidney. Sci. World J..

[58] Hilliard L.M., Nematbakhsh M., Kett M.M., Teichman E., Sampson A.K., Widdop R.E., Evans R.G., Denton K.M. (2011). Gender differences in pressure-natriuresis and renal autoregulation: Role of the angiotensin type 2 receptor. Hypertension.

[59] Ikebuchi Y., Masumoto N., Tasaka K., Koike K., Kasahara K., Miyake A., Tanizawa O. (1991). Superoxide anion increases intracellular pH, intracellular free calcium, and arachidonate release in human amnion cells. J. Biol. Chem..

[60] Hu Q., Xia Y., Corda S., Zweier J.L., Ziegelstein R.C. (1998). Hydrogen peroxide decreases pH_i_ in human aortic endothelial cells by inhibiting Na^+^/H^+^ exchange. Circ. Res..

[61] Pinelli L., Nissant A., Edwards A., Lourdel S., Teulon J., Paulais M. (2016). Dual regulation of the native ClC-K2 chloride channel in the distal nephron by voltage and pH. J. Gen. Physiol..

[62] Gradogna A., Babini E., Picollo A., Pusch M. (2010). A regulatory calcium-binding site at the subunit interface of CLC-K kidney chloride channels. J. Gen. Physiol..

[63] Paulais M., Bloch-Faure M., Picard N., Jacques T., Ramakrishnan S.K., Keck M., Sohet F., Eladari D., Houillier P., Lourdel S., Teulon J., Tucker S.J. (2011). Renal phenotype in mice lacking the K_ir_5.1 (Kcnj16) K^+^ channel subunit contrasts with that observed in SeSAME/EAST syndrome. Proc. Natl. Acad. Sci. U. S. A..

[64] Hunyady L., Catt K.J. (2006). Pleiotropic AT1 receptor signaling pathways mediating physiological and pathogenic actions of angiotensin II. Mol. Endocrinol..

[65] McCallum L., Lip S., Padmanabhan S. (2015). The hidden hand of chloride in hypertension. Pflugers Arch..

[66] Kotchen T.A., Galla J.H., Luke R.G. (1976). Failure of NaHCO_3_ and KHCO_3_ to inhibit renin in the rat. Am. J. Physiol..

[67] Kotchen T.A., Luke R.G., Ott C.E., Galla J.H., Whitescarver S. (1983). Effect of chloride on renin and blood pressure responses to sodium chloride. Ann. Intern. Med..

[68] Luft F.C., Steinberg H., Ganten U., Meyer D., Gless K.H., Lang R.E., Fineberg N.S., Rascher W., Unger T., Ganten D. (1988). Effect of sodium chloride and sodium bicarbonate on blood pressure in stroke-prone spontaneously hypertensive rats. Clin. Sci..

[69] Burson J.M., Aguilera G., Gross K.W., Sigmund C.D. (1994). Differential expression of angiotensin receptor 1A and 1B in mouse. Am. J. Physiol..

[70] Zaika O., Tomilin V.N., Pochynyuk O. (2020). Adenosine inhibits the basolateral Cl^-^ ClC-K2/b channel in collecting duct intercalated cells. Am. J. Physiol. Renal Physiol..

[71] Tomilin V.N., Mamenko M., Zaika O., Ren G., Marrelli S.P., Birnbaumer L., Pochynyuk O. (2019). TRPC3 determines osmosensitive [Ca^2+^]_i_ signaling in the collecting duct and contributes to urinary concentration. PLoS One.

