# Supplementary material for: Angiotensin II increases activity of the ClC-K2 Cl− channel in collecting duct intercalated cells by stimulating production of reactive oxygen species
Source: J Biol Chem. 2021 Jan 30;296:100347. doi: 10.1016/j.jbc.2021.100347 (PMC7949157; doi:10.1016/j.jbc.2021.100347)
Supplement: Supplemental Figures S1–S2 [file mmc1.pdf]

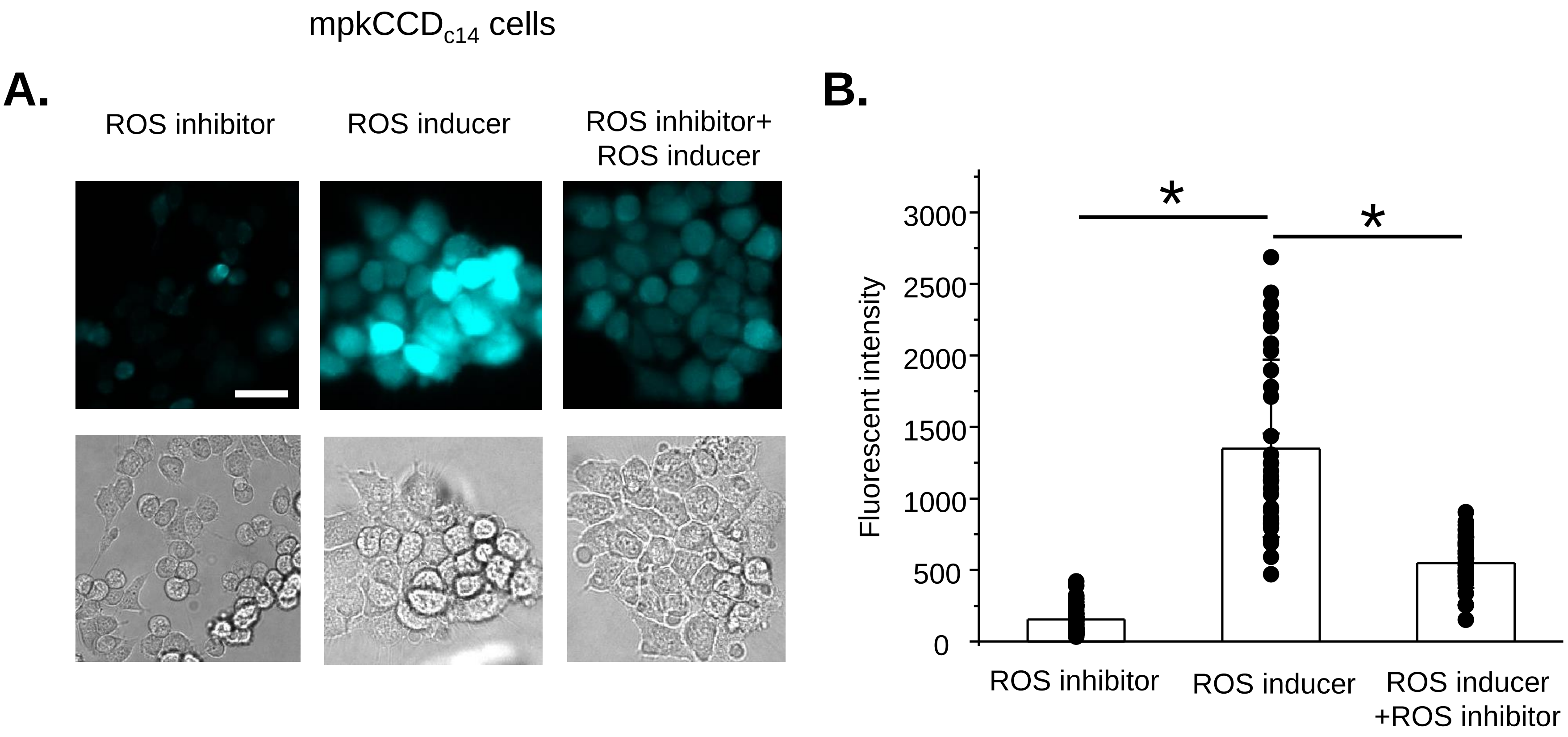

<u>Figure S1.</u> **(A)** Representative micrographs of subconfluent mpkCCD$_{c14}$ cells loaded with the oxidative stress detection reagent to report ROS levels upon treatment with ROS inhibitor, N-acetyl-L-cysteine (10mM for 30 min), ROS inducer pyocyanin (200 μM for the last 20 min), and concomitant treatment with both agents. All images were captured with identical intensity and exposure settings. Scale bar is 50 μm. **(B)** Summary graph of intensities of ROS-reporting fluorescent signals in individual mpkCCD$_{c14}$ cells for the conditions shown in (A). Both SEM (smaller bars) and SD (larger bars) are shown for each tested condition. * - significant changes between groups ($P < 0.05$) are shown with respective lines on top (one-way ANOVA).

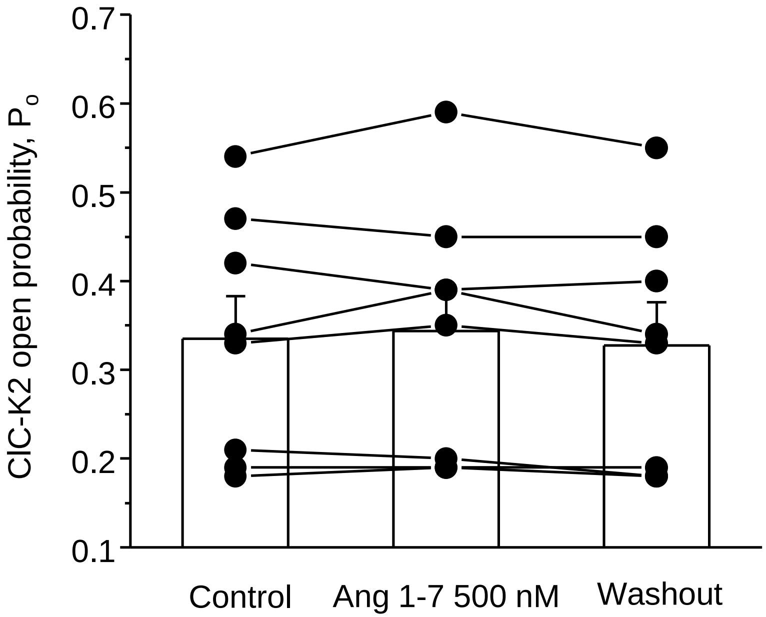

Figure S2. Summary graph of changes in ClC-K2 open probability (P_o) in patch clamp experiments on the basolateral membrane of intercalated cells in the control, upon treatment with 500 nM Ang 1-7, and following washout with control medium.
